# Physician’s Knowledge of Appropriate Prescribing for the Elderly—A Survey Among Family and Internal Medicine Physicians in Nigeria

**DOI:** 10.3389/fphar.2019.00592

**Published:** 2019-05-31

**Authors:** Joseph O. Fadare, Abimbola Margaret Obimakinde, Okezie O. Enwere, Olufemi O. Desalu, Raphael Olasoji Ibidapo

**Affiliations:** ^1^Department of Pharmacology and Therapeutics, Ekiti State University College of Medicine, Ado-Ekiti, Nigeria; ^2^Family Medicine Unit, Department of Community Medicine, College of Medicine, University of Ibadan, Ibadan, Nigeria; ^3^Family Medicine Department, University College Hospital, Ibadan, Nigeria; ^4^Department of Medicine, Imo State University, Orlu, Nigeria; ^5^Department of Medicine, College of Health Sciences, University of Ilorin, Ilorin, Nigeria; ^6^Department of Medicine, Ekiti State University Teaching Hospital, Ado-Ekiti, Nigeria

**Keywords:** inappropriate prescribing, elderly patients, rational prescribing, adverse drug reactions, physicians

## Abstract

**Background:** Prescription and use of inappropriate medications have been identified as a major cause of morbidity among the elderly. Several screening tools have been developed to identify inappropriate medications prescribed for elderly patients. There is dearth of information about the knowledge of Nigerian physicians regarding these screening tools and appropriate prescribing for the elderly in general. The primary objective of this study was to assess the knowledge of Nigerian physicians about these screening tools and appropriate prescribing of medications for the elderly.

**Methods:** The study was a cross-sectional questionnaire-based study conducted among physicians working in Family Medicine and Internal Medicine departments of four tertiary health care facilities in Nigeria. The questionnaire consisted of sections on general characteristics of respondents and their knowledge of four selected screening tools for inappropriate medications in the elderly. Ten clinical vignettes representing different therapeutic areas (using the best option type questions) about medicine use in the elderly were included with a score of 1 and 0 for correct and wrong answers, respectively. The knowledge of respondents was classified as (total score, over 10): poor (score, < 5), average (score, 5-6), and good (score, 7-10).

**Results:** One hundred and five physicians returned completed questionnaires. Twenty percent of respondents knew about Beers criteria, whereas 15.6% were familiar with the STOPP criteria. Majority (83; 84.7%) of the respondents were confident of their ability to prescribeappropriately for elderly patients. The mean knowledge score was 5.3 ± 2.0 with 32 (30.5%), 41 (39%), and 32 (30.5%) having low, average, and good scores, respectively. The association between the knowledge score, duration of practice, and seniority was statistically significant (OR, 3.6, *p* = .004 and OR, 3; *p* = .012), respectively.

**Conclusion:** There are significant gaps in the knowledge of Nigerian physicians about screening tools for inappropriate medications. There is a need for stakeholders involved in the care of elderly Nigerian patients to develop new strategies to improve services being offered. These may include introduction of modules on appropriate prescribing in the curriculum of undergraduate and postgraduate medical education and the routine use of some screening tools for inappropriate medications in daily clinical practice.

## Introduction

Drug treatment of elderly patients is associated with potential adverse drug reactions (ADRs) with prescription and use of inappropriate medications being responsible in many cases (Dormann et al., 2013; Oscanoa et al., 2017). A systematic review on the prevalence and risk factors for ADRs in the elderly found a mean prevalence of 10% (Alhawassi et al., 2014). Patel and Patel (2018) demonstrated a four-fold increase in mortality due to ADR in elderly patients when compared to children. Identified factors for ADR in the elderly include polypharmacy and multiple co-morbidities on the background of declining physiological functions of the liver and kidneys (Joshua et al., 2007; Alhawassi et al., 2014; Sonnerstam et al., 2016). Also, changes in cognitive functions may affect adherence and other medicine-use issues (Wucherer et al., 2017).

Several tools/criteria have been developed over time to address the problem of inappropriate prescribing for elderly patients and these include the Beers criteria, Screening Tool of Older Persons’ potentially inappropriate Prescription (STOPP) criteria, Medication Appropriateness Index (MAI), and the Zhan criteria (Beers et al., 1991; Hanlon et al., 1992; Zhan et al., 2001; Gallagher et al., 2008). Other recently developed tools are the European Union list of potentially inappropriate medications (EU(7)-PIM list) (Renom-Guiteras et al., 2015), PRISCUS (developed in Germany) (Holt et al., 2010), and Improved Prescribing in the Elderly Tool (IPET) (Naugler et al., 2000) developed in Canada. Emerging economies like Brazil and Thailand have also developed their own context-specific instruments to screen for inappropriate medications in the elderly building on the foundation of the older instruments (Almeida et al., 2018; Prasert et al., 2018). The Beers list, an explicit criterion initially developed in 1991, has been regularly updated to cater for new scientific findings regarding appropriateness of medications in the elderly. Its latest update was in 2015 by the American Geriatric Society (AGS), and most of the newer tools were developed using it as a model (American Geriatrics Society, 2015). Negative patient outcomes, such as falls and delirium, have been associated with some of the drugs listed in the Beers criteria (Rothberg et al., 2013; Bazargan et al., 2018). The main emphasis of the STOPP criteria is in the area of avoidable adverse drug events (ADEs) and potential drug–drug interactions. Results from a meta-analysis of four randomized control trials (RCTs) showed a reduction in falls and episodes of delirium when STOPP criteria were applied (Hill-Taylor et al., 2016). The MAI is a 10-item question-based implicit criterion that takes into consideration the indication, effectiveness, dose, and direction for use among others (Hanlon and Schmader, 2013). The Zhan criteria, a modification of the Beers criteria, classify into 33 drugs into three categories according to their appropriateness or otherwise (Zhan et al., 2001).

The body of research in the area of prescription of potentially inappropriate medications and its consequences in elderly patients continues to grow in developing countries, like Nigeria and South Africa (Fadare et al., 2013; Fadare et al., 2015; Van Heerden et al., 2016; Akande-Sholabi et al., 2018; Saka et al., 2018). With improvement in the management of communicable diseases, such as HIV/AIDS, malaria, and tuberculosis, people are living longer in many developing countries and as such the proportion of elderly patients is expected to rise significantly in the next decade. Nigeria, the most populous country in Africa, has an estimated population of over 190 million with the elderly comprising just over 5% [Nigerian Demographic and Health Survey (NDHS), 2013]. The health care system is made up of private, faith-based, and public health care facilities. The public health care system of the country currently comprises three levels of care: primary, secondary, and tertiary. The tertiary level facilities, which are made up of Federal Medical Centres and University Teaching Hospitals, are the best equipped and have the most qualified health care personnel in the country. They are also training centers for medical students and postgraduate resident doctors in various specialties of medicine. These centers are the most patronized by the public because of the poor level of functionality of many primary and secondary level facilities in Nigeria. Therefore, there is a need for prescribers to be knowledgeable about potentially inappropriate medications and to actively screen prescriptions written for elderly patients. There are reports from some countries about the knowledge and attitude of physicians toward prescription of potentially inappropriate medications for elderly (Maio et al., 2011; Ramaswamy et al., 2011). There is, however, a dearth of information regarding this important issue in Nigeria and thus is the rationale for this study.

The primary objective of this study was to assess the knowledge of Nigerian physicians working in Family Medicine and Internal Medicine about appropriate prescribing of medications for the elderly. Secondary objectives included familiarity with screening tools for inappropriate medications and identification of barriers limiting appropriate prescription for the elderly.

## Methodology

The study was a cross-sectional questionnaire-based study conducted among physicians working in four tertiary health care facilities in Nigeria.

## Study Setting

The study was conducted in the Family and Internal Medicine departments of four tertiary level health care facilities located in the South-Western, South-Eastern, and North-Central regions of Nigeria between January and April 2017. The health care facilities are: Ekiti State University Teaching Hospital, Ado-Ekiti (South-West), University College Hospital, Ibadan (South-West), University of Ilorin Teaching Hospital, Ilorin (North-Central), and the Imo State University Teaching Hospital, Orlu (South-East). The choice of these two specialities is because elderly outpatients are seen mainly in the clinics manned by residents and consultants from the departments. The departments of family and internal medicine in each of these facilities run daily outpatient clinics for ambulatory patients. The participating centers were selected through convenient sampling as some of the co-investigators were based there.

## Study Participants

### Inclusion Criteria

Physicians (residents and consultants) working in the Family and Internal Medicine Departments of the participating health care facilities who consented to the study.

### Exclusion Criteria

House physicians working in the Family and Internal Medicine Departments of the participating health care facilities because of their limited knowledge and experience with prescribing.

## Study Instrument

The self-administered questionnaire, developed in English language, was adapted from similar studies conducted in Italy and India among primary care physicians (Maio et al., 2011; Ramaswamy et al., 2011) and consisted of two parts: bio-demographics details, prescription experience for the elderly and respondent’s perception about factors affecting appropriate prescribing for the elderly are captured in the first section. The second part was made up of 10 clinical vignettes about medication use in the elderly in the form of best option multiple-choice questions with a score of 1 and 0 allocated for a correct and wrong answer, respectively (**Supplementary Material Appendix A**). The scenarios described in the vignettes consisted of therapeutic problems of the central nervous (Nos. 3, 6, 10), endocrine (No. 9), musculoskeletal (No. 5), and cardiovascular systems (Nos. 1, 2, 7, 8). The knowledge of respondents was classified as (total score over 10): poor (score <5), average (score 5–6), and good (score 7–10). The questionnaire was reviewed for reliability and content validity by the principal researcher and two co-authors who are experts in clinical pharmacology and therapeutics, geriatrics and family medicine. Subsequently, it was pilot tested among 10 physicians working in the Family Medicine Department of a tertiary health care facility located within the same region, which was not part of the study. Based on their responses, the questionnaire was revised and necessary adjustments made before administration to study participants.

All physicians working in the departments of Family Medicine and Internal Medicine of the four participating hospitals were approached to participate in the study. The chief residents of the selected departments were asked to distribute the questionnaires to the physicians during mandatory departmental programs such as grand rounds and seminars. A time frame of 30 min was allowed for the completion of the questionnaire.

## Sample Size

A convenience sampling method that included all 142 physicians working in the Family Medicine and Internal Medicine Departments of four tertiary health care facilities in three geo-political areas of the country was used for this study. All the 142 physicians were invited to participate in the study.

## Ethical Consideration

Ethical approval was obtained from the Research and Ethics Committee of the Ekiti State University Teaching Hospital, Ado-Ekiti, Nigeria before commencement of the study. Because the study was questionnaire-based and noninvasive, permission was granted by the other centers based on approval from the primary site.

## Statistical Analysis

The information obtained from the questionnaire was coded, entered, and analyzed using IBM SPSS version 19. Analysis was done using descriptive statistics, which was used to obtain the general characteristics of the study participants. Chi-square was used to determine the level of significance of groups of categorical variables. P values < 0.05 were considered significant.

## Results

### Socio-Demographic Details

One hundred and five physicians returned the completed questionnaires for analysis—a response rate of 73.9%. Majority of respondents (69; 65.7%) were males, senior medical doctors, and worked in internal medicine (65.7%, 52.8%, and 57.3%, respectively). **Table 1** shows the demographic characteristics of respondents. The mean duration of practice was 8.3 ± 6.7 years with 23.8%, 45.7%, and 30.5% of the respondents having practiced for <5 years, 5 to 10 years, and more than 10 years, respectively.

**Table 1:** Demographic details of respondents.

Variable	Frequency (%)
Sex	
Male	69 (65.7%)
Female	36 (34.3%)
Position of respondent	
Medical officer	19 (18.1)
Junior resident	31 (29.6)
Senior resident	43 (41)
Consultant	12 (11.4)
Specialty	
Internal medicine	59 (56.2)
Family medicine	46 (43.8)
Duration of practice	
1–5 years	40 (38.1)
>5 years	65 (61.9)

### Respondents’ Knowledge of Screening Tools for Inappropriate Medications and Sources of Drug Information

**Figure 1:**
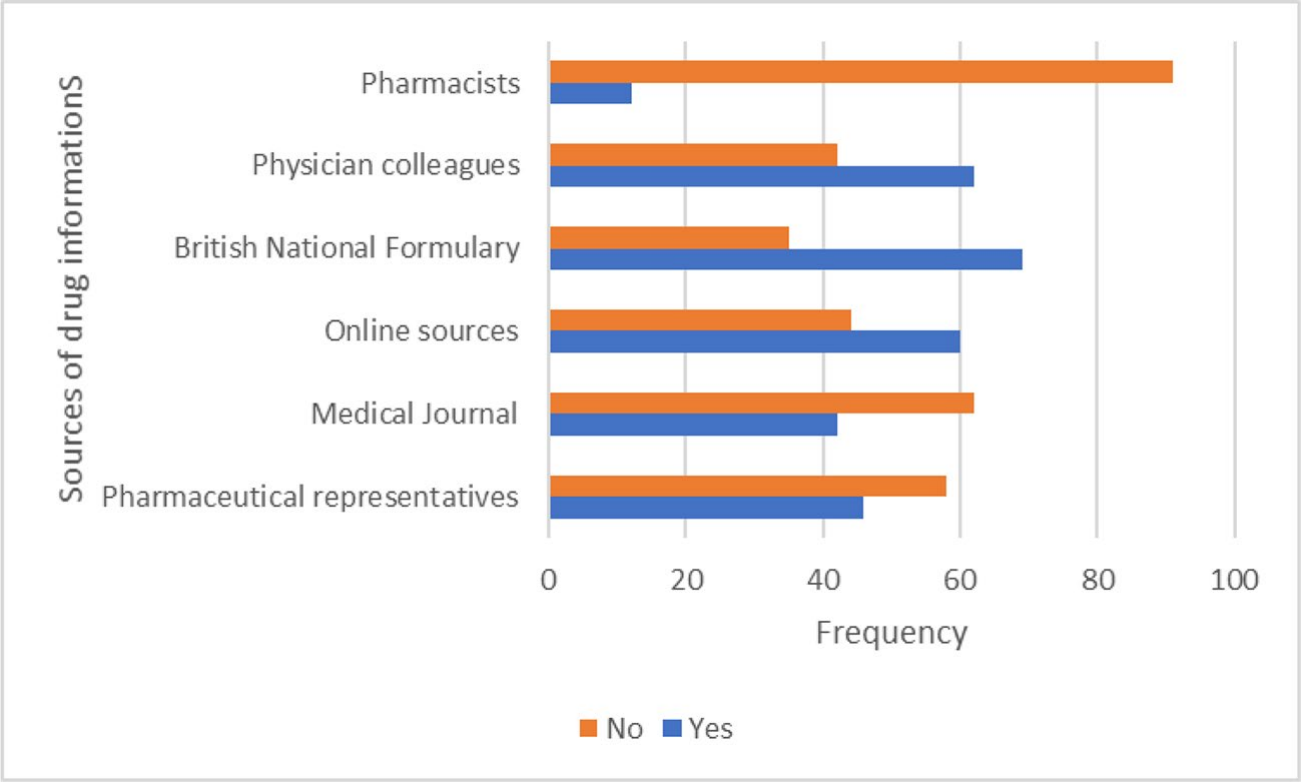
The sources of drug information used by respondents.

**Figure 2:**
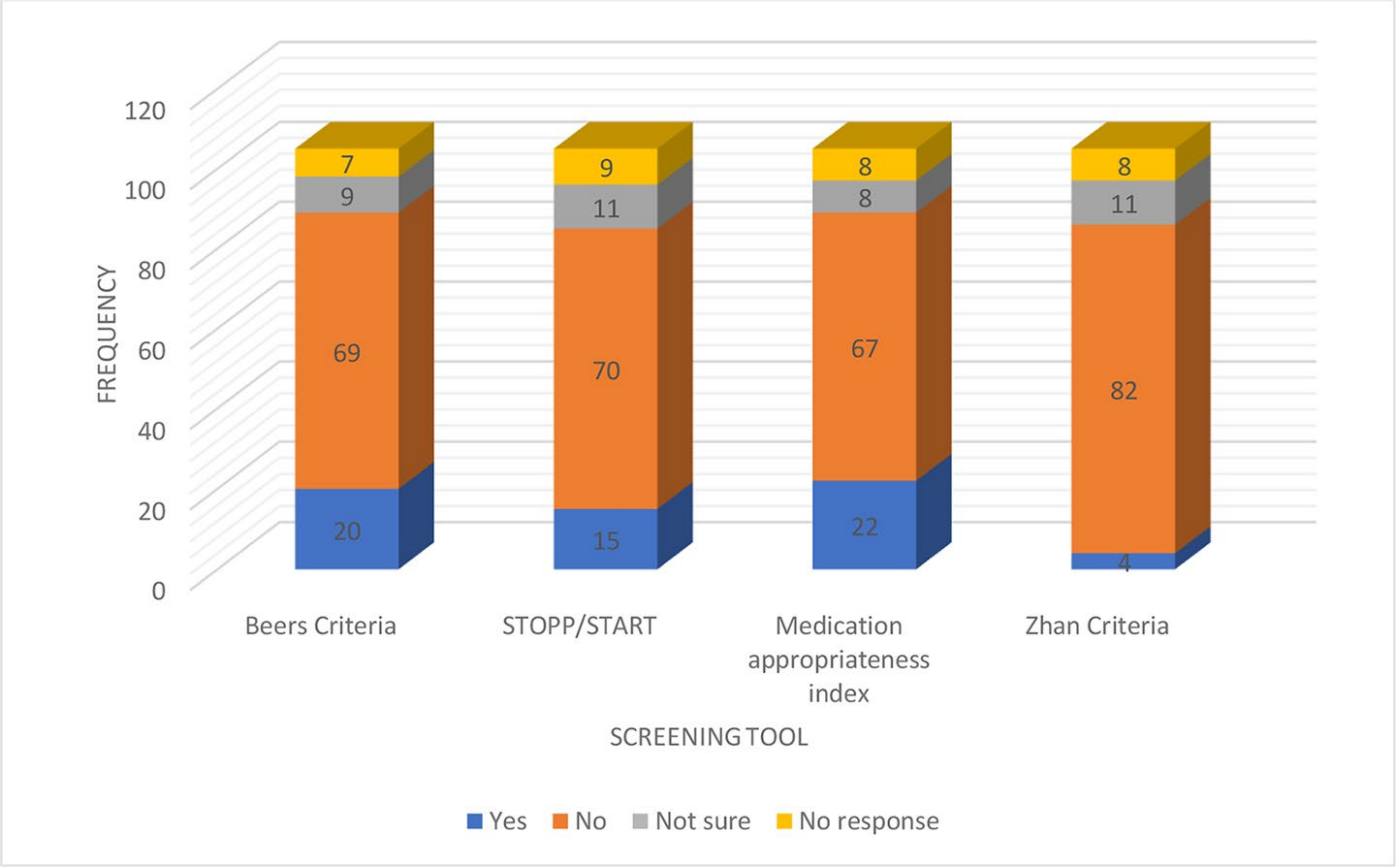
Respondents knowledge of the screening tools.

The sources of drug information used by respondents were the British National Formulary (BNF), consultations with colleagues, and online sources (**Figure 1**). Regarding respondents’ knowledge about screening tools for inappropriate medications, 20.4% and 22.7% of them knew about Beers criteria and MAI, while 15.6% were familiar with the STOPP criteria. **Figure 2** highlights respondents’ knowledge of the screening tools. Majority (83; 84.7%) of the respondents were confident of their ability to prescribe rationally for elderly patients.

### Barriers Against Appropriate Prescribing for the Elderly

Multiple medications (81; 82.6%), potential drug interactions (79; 81.4%), and cost of medicines to the patients (80; 80%) were the most commonly identified barriers against appropriate prescribing for the elderly (**Table 2**).

**Table 2:** Identified barriers against appropriate prescribing for the elderly.

Factors	Strongly agree (%)	Agree (%)	Neutral (%)	Disagree (%)	Strongly disagree (%)	Missing
Lack of time in the office schedule	12 (12.1)	21 (21.2)	18 (18.2)	35 (35.4)	13 (13.1)	6
Lack of acceptable therapeutic alternatives	5 (5.2)	45 (46.4)	20 (20.6)	21 (21.6)	6 (5.7)	8
Potential drug interactions	24 (24.7)	55 (56.7)	9 (9.3)	7 (7.2)	2 (2.1)	8
Cost of medication to patient	28 (28)	52 (52)	10 (10)	8 (8)	2 (2)	5
Patient request to begin a specific medication	4 (4.1)	29 (29.6)	30 (30.6)	30 (30.6)	4 (4.1)	8
Lack of information about which medications a patient is already taking	5 (5.1)	56 (57.1)	11 (11.2)	24 (24.5)	2 (2)	7
Lack of formal education on prescribing for the elderly	8 (0)	33 (33)	18 (18)	33 (33)	8 (8)	5
Large number of medications a patient is taking	21 (21.2)	61 (61.6)	6 (6.1)	10 (10.1)	1 (1)	6
Unwillingness to discontinue a medication prescribed by another physician	3 (3.1)	34 (34.7)	26 (26.5)	34 (34.7)	1 (1)	7
Difficulty communicating with other physicians who participate in a patient’s care	10 10.1)	37 (37.4)	20 (20.2)	30 (30.3)	2 (2)	6
No feedback from the pharmacy	21 (21.4)	34 (34.7)	21 (21.4)	19 (19.4)	3 (3.1)	7

### Respondents’ Knowledge of Presented Case Vignettes and Association With Other Study Variables

The mean knowledge score was 5.3 ± 2.0 with 32 (30.5%), 41 (39%), and 32 (30.5%) having low, average, and good scores, respectively. Overall, most respondents had good knowledge of appropriate use of medicines in the elderly in the following therapeutic areas: non-steroidal anti-inflammatory drugs (NSAIDs) (90; 87.6%), centrally acting antihypertensives in the elderly (92; 87.6% and 76; 76.4%), the use of first generation sulfonylureas (81; 77.1%), and the correct use of Nifedipine in hypertensive emergency (80; 76.2%). Poor level of knowledge was demonstrated in therapeutic areas of insomnia (19; 18.1%) and depression (25; 27.2%). The clinical scenario on the effect of NSAIDs on heart failure was answered correctly by 61 (58.1%) respondents.

The variables duration of practice, position, and knowledge score were dichotomized for ease of inferential analysis into the following: younger and older than 5 years, junior (junior residents and medical officers) and senior (senior residents and consultants), and score less than 5 and greater than 5. **Table 3** shows that the association between the knowledge score, duration of practice, and seniority was statistically significant. The strength of this association is further confirmed by the odds ratios (OR) of 3.6 and 3, respectively.

**Table 3:** Association between the knowledge score and some variables.

Variables	Score 0–4	Score 5–10	Chi-square	OR	95% CI
Junior physician	21	28	.012*	3.0*	1.26–7.16
Senior physician	11	44			
1–5 years practice	19	21	.004*	3.62*	1.52–8.63
>5 years practice	13	52			
Male	20	49	.646	0.82	0.34–1.94
Female	12	24			
Family medicine	15	29	.67	0.78	0.33–1.81
Internal medicine	17	42			

*The variables duration of practice, position, and knowledge score were dichotomized for ease of inferential analysis into the following: less than or greater than 5 years, junior (junior residents and medical officers), and senior (senior residents and consultants).

## Discussion

This study assessed the knowledge of physicians working in Internal and Family Medicine departments regarding appropriateness of medicines for the elderly and its screening tools. Overall, the knowledge score of respondents was above average with majority of them dealing correctly with clinical scenarios affecting the cardiovascular, endocrine, and gastrointestinal systems.

Screening tools, such as the Beers criteria, STOPP, MAI, and Zhan criteria, are a group of explicit and sometimes implicit classification of medicines used for elderly patients. Medications on these lists have been found to contribute significantly to morbidity in elderly patients (Price et al., 2014; Sarwar et al., 2018). Only about a fifth of respondents in this study knew about the Beers criteria and the MAI with another 15% having information about the STOPP criteria. The knowledge level among respondents in our study is slightly better than that reported in qualitative studies among general practitioners in Australia and Germany (Magin et al., 2015; Pohontsch et al., 2017). In the just-cited studies, respondents showed a high level of confidence in appropriate prescribing for elderly patients despite their poor knowledge of the screening tools. Similarly, over 80% of our respondents were also confident of appropriate prescribing for the elderly despite their poor knowledge of the screening tools. This might be due to the fact that many physicians may have used their residual knowledge of clinical pharmacology and understanding of the clinical scenarios to address prescribing issues in the elderly.

Respondents in this study identified high cost of medicines, potential drug interactions, and difficulty in communicating with other prescribers as barriers in addition to the ones cited in literature. Barriers to appropriate prescribing for the elderly from literature include limited knowledge of potentially inappropriate medications, extra time needed to consult the criteria, high number of prescribed medications, poor communication, and lack of formal education of prescribing guidelines (Maio et al., 2011; Clyne et al., 2016; Voigt et al., 2016).

Close to 70% of respondents scored above average in the clinical vignettes, indicating a good understanding of therapeutic issues in elderly patients. Our findings are similar to that from an Italian study with seven clinical scenarios where only 17% of respondents had low scores (Maio et al., 2011). Similarly, the mean score in an Indian study among family and internal medicine physicians was above average (3.9/6) (Ramaswamy et al., 2011). Respondents in our study were more knowledgeable in therapeutic areas of hypertension, diabetes mellitus, and osteoarthritis. This is not surprising because these are the most common disease conditions found among Nigerian elderly patients (Fadare et al., 2013, Fadare et al., 2015; Cadmus et al., 2017). This is in contrast to areas like arrhythmias, insomnia, depression, and other central nervous system (CNS) problems where low scores were recorded. The poor knowledge in therapeutic areas of insomnia and depression also resonates with the findings from Nigerian studies on prescription of inappropriate medicines where benzodiazepines (diazepam) and antidepressants (amitriptyline) were among the most common inappropriate medicines identified (Fadare et al., 2013,Fadare et al., 2015; Akande-Sholabi et al., 2018). On the contrary, respondents in the earlier-cited Italian study had better than average knowledge in therapeutic areas of insomnia and depression, whereas a similar poor performance was reported in the clinical vignette on the use of amiodarone in the elderly (Maio et al., 2011). The association between the knowledge score, duration of practice, and position was strong with OR of 3.62 and 3, respectively. In contrast, the study by Maio et al. (2011)reported respondents with low score had been in practice for longer duration. The reported higher score in our study is likely due to the working environment of respondents with many of them at various levels of postgraduate specialist training or were consultant physicians in charge of the training programs.

The BNF, physician colleagues, and the Internet were the most common sources of medicines information used by respondents. Medical textbooks, journals, and physician colleagues were the most identified sources in an earlier study conducted in Italy (Maio et al., 2011). Similarly, report from an earlier Nigerian survey among 163 medical doctors indicated information from colleagues, reference books, and pharmaceutical sales representatives as the main sources (Oshikoya et al., 2011). The rapid growth of information and communication technology (ICT) globally and especially its use in medicine is likely responsible for the change in trend.

## Study Limitations

This study is associated with some limitations. The study was conducted in a few tertiary health care facilities and hence may not reflect the reality among physicians working in primary and secondary care facilities worldwide nationwide. Since the questionnaire was also self-administered, there is also the possibility of bias while responding. The lack of similar studies from Nigeria and other African countries with which to compare our findings may also have affected the discussion of this study. However, the use of previously validated questionnaire, relatively high proportion of respondents from the sampling frame, representation from different cadres of physicians and different regions of the country are potential strengths of the study.

## Conclusion

There are significant gaps in the knowledge of Nigerian physicians about screening tools for inappropriate medications. There is a need for stakeholders involved in the care of elderly Nigerian patients to develop new strategies to improve services being offered. This may include introduction of modules on appropriate prescribing in the curriculum of undergraduate and postgraduate medical education in Nigeria.

## Implications for Practice (Future Directions)

There is a need to incorporate the use of some of the established screening tools, such as the Beers and STOPP criteria in the daily clinical practice of Nigerian physicians. The development of a context-specific screening tool may be the direction to go for stakeholders in geriatric care in Nigeria.

## Authors Contributions

JF conceptualized and designed the study. He also analyzed the data and developed the first draft of the manuscript. AO took active part in conceptualizing the study. She was involved in data acquisition and analysis and reviewed the initial draft of manuscript for critical intellectual content. OE was involved in conducting the literature search for the work and also in data acquisition, and reviewed the initial draft of the manuscript. OD was involved in data acquisition and analysis and reviewed the draft manuscript before final approval. RI was involved in the literature search, data acquisition, and analysis, and also reviewed the draft manuscript. All co-authors approved the final draft of the manuscript.

## Conflict of Interest Statement

The authors declare that the research was conducted in the absence of any commercial or financial relationships that could be construed as a potential conflict of interest.
